# Urolithin A reduces amyloid-beta load and improves cognitive deficits uncorrelated with plaque burden in a mouse model of Alzheimer’s disease

**DOI:** 10.1007/s11357-022-00708-y

**Published:** 2022-12-28

**Authors:** Josué Ballesteros-Álvarez, Wynnie Nguyen, Renuka Sivapatham, Anand Rane, Julie K. Andersen

**Affiliations:** Buck Institute for Research on Aging, Novato, CA USA

**Keywords:** AD, Aβ, Cognition, Urolithin A, Autophagy, Lifespan

## Abstract

In the present study, we investigated the effects of urolithin A (UA), a metabolite generated from ellagic acid via its metabolism by gut bacteria, as an autophagy activator with potential neuroprotective activity. WT and 3xTg-AD mice were administered long-term intermittent dietary supplementation with UA. UA was found to prevent deficits in spatial memory, cued fear response, and exploratory behavior in this model. It also decreased the Aβ plaque burden in areas of the hippocampus where these protein deposits are prominent in the model. Interestingly, correlation analyses demonstrate that Aβ plaque burden positively correlates with enhanced spatial memory in 3xTg-AD mice on a control diet but not in those supplemented with UA. In contrast, Aβ42 abundance in cortical and hippocampal homogenates negatively correlate with spatial memory in UA-fed mice. Our data suggest that plaque formation may be a protective mechanism against neurodegeneration and cognitive decline and that targeting the generation of proteotoxic Aβ species might be a more successful approach in halting disease progression. UA was also found to extend lifespan in normal aging mice. Mechanistically, we demonstrate that UA is able to induce autophagy and to increase Aβ clearance in neuronal cell lines. In summary, our studies reveal UA, likely via its actions as a autophagy inducer, is capable of removing Aβ from neurons and its dietary administration prevents the onset of cognitive deficits associated with pathological Aβ deposition in the 3xTg-AD mouse model as well as extending lifespan in normal aging mice.

**Supplementary information:**

The online version contains supplementary material available at 10.1007/s11357-022-00708-y.

## Background

Alzheimer’s disease (AD) is the most common form of dementia and an increasingly critical public health issue, particularly in countries where life expectancy is higher [1]. The two most well-known neuropathological events that occur during AD pathogenesis are the deposition of amyloid beta (Aβ) plaques and tau protein neurofibrillary tangles, particularly in the hippocampus [2, 3].

AD-associated amyloid plaques contain amyloid beta peptides, a protein consisting of 36–43 amino acids that is generated when the amyloid precursor protein (APP) is sequentially cleaved by beta and gamma secretases [4, 5]. One of the core proteins of the gamma secretase complex is the presenilin protein (PSEN); familial mutations in the PSEN1 gene have been associated with increased Aβ production [6]. Aβ40 and Aβ42 are the two most common cleaved peptides that are generated; whereas Aβ40 is the most abundant overall, Aβ42 is believed to configure the bulk of the AD amyloid plaque formation and to be the main contributor to neuronal toxicity and neuropathology [7, 8]. Importantly, recent scientific evidence suggests that soluble oligomeric forms of Aβ may drive AD neuropathology to a greater extent than the more conspicuous plaque-forming insoluble fibrils [9–11].

A growing body of scientific evidence suggests that gut dysbiosis may play an important role in the development of age-related neurodegenerative disorders including AD [12–15], a phenomenon referred to as the “gut-brain axis hypothesis” [16, 17]. This hypothesis, which has become a matter of growing scientific interest in the field, proposes that endogenously produced gut metabolites are able to modulate a myriad of biological processes fundamental for neuronal homeostasis. One such metabolite is urolithin A (UA). UA is produced in the colon when gut bacteria break down ellagitannins, natural compounds which are found in edible plants such as pomegranates, strawberries, and walnuts [18]. Numerous studies have shown that UA robustly improves mitochondrial activity, muscle function, lifespan, and cognition in several animal models [19–21]. Importantly, the metabolite has been shown to be safe for oral administration in clinical trial in aging humans, where it was shown to preserve muscle health [22, 23].

Neurons have high energetic demands that rely on the preservation of mitochondrial homeostasis, which requires functional mitophagy and mitochondrial remodeling [24]. Mitophagy consists of the selective degradation of mitochondria by the autophagic machinery [25], tightly linking the two biological processes, mitophagy, and the broader macroautophagy. Whereas recent studies have strongly suggested mitophagy induction as underlying the beneficial effects of UA [19–22, 26], questions remain regarding UA’s involvement in lysosome-mediated clearance of substrates beyond mitochondria including of characteristic neurotoxic protein species.

In this present study, we evaluated cognitive function in 12-to-13-month-old female 3xTg-AD mice and the impact of UA dietary intervention on observed deficits. For this purpose, we used a battery of behavioral tests in order to assess spatial learning and memory, associative learning, willingness to explore, locomotor activity, and working memory. Here, we report that the compound reduced Aβ burden and enhanced cognition in the 3xTg-AD mice. UA was also able to increase lifespan in normal aging wild-type mice and to enhance autophagic Aβ clearance in neuronal cell types in culture.

## Materials and methods

### Reagents and antibodies

Urolithin A was purchased from Cayman Chemical Company (#22,607). Bafilomycin-A1 (#B1793) and anti-p62/SQSTM1 antibody (#P0067) were purchased from Sigma-Aldrich. Anti-amyloid beta peptide antibody (MOAB-2) was purchased from Millipore (#MABN254). AT8 phospho-Tau antibody (Ser202, Thr205) was purchased from Thermo Fisher Scientific (#MN1020). Anti-β-amyloid antibody (6E10) (#803,017) was purchased from Biolegend. LC3 antibody was purchased from Proteintech (#14,600–1-AP). Actin antibody was purchased from Cell Signaling Technology (#3700) goat anti-mouse IgG (H + L) cross-adsorbed secondary antibody, Alexa Fluor™ 488, was purchased from Sigma-Aldrich (#A-11001). DMEM/F12 (#11,330,032) and DQ-BSA red (#D12051) were purchased from Thermo Fisher Scientific. Earl’s balanced salt solution (EBSS) (#24,010–043) was purchased from Gibco. DMEM (#10–013-CV), fetal bovine serum (#35–010-CV) and penicillin–streptomycin solution (#30–002-CI) were purchased from Corning. Beta amyloid oligomers were generated according to the manufacturer’s instructions using the beta amyloid (1–42), aggregation kit (#A-1170–2) purchased from rPeptide.

### Animals and treatment

All mice husbandry and experimental procedures were practiced in accordance to protocols approved by the Buck’s Institutional Animal Care and Use Committee (IACUC).

Female 3xTg-AD [Tg(APPSwe,tauP301L)1Lfa Psen1/Mmjax] (#034,830) [27] transgenic mice, female B6129SF2/J (#101,045), and male C57BL/6NJ (#005,304) wild-type mice were purchased from the Jackson Laboratory. Starting at 3 months of age and until the noted experimental endpoints, mice were fed regular chow or chow supplemented with 25 mg/kg of UA in alternate weeks (1 week on, 1 week off). Estimated oral consumption of UA is 5 mg kg day^−1^. Food pellets were purchased from Dyets (#AIN-93G) and UA from Cayman Chemical Company (#22,607). All animals had free access to food and water. 3xTg-AD mice or control B6129SF2/J mice were used for behavioral analysis at 12–13 months of age and then euthanized for biochemical and neuropathological analysis.

### Immunofluorescence of mouse brain tissue

Anesthetized mice were perfused with 1 × PBS and whole brains were sagittally bisected. Left hemispheres were placed in 4% PFA for 48 h, then washed in cold TBS and cut using a Leica VT 1000S Vibratome. Fifty-micrometer coronal sections were obtained and stored in cryogenic buffer (0.1 M potassium acetate, 40% ethylene glycol, and 1% polyvinyl pyrrolidone) at − 20 °C. Free-floating sections showcasing the rostral hippocampus were subject to epitope unmasking in 88% formic acid for 3 min, then transferred to 500-μL citrate buffer (0.01 M, 6.0 pH) and microwaved 3 × 5 s at 100% power. Sections were then washed 3 × in TBS, permeabilized for 1 h in TBS plus 0.2% Triton X-100, blocked for 1 h in 5% normal goat serum in TBS 0.2% Triton X-100 permeabilization buffer for 1 h. Subsequently, sections were incubated in blocking buffer at 4 °C overnight with anti-amyloid beta peptide antibody (MOAB-2) (1:200) or at room temperature overnight with anti-phospho-Tau AT8 antibody (1:50). Sections were washed 3 × with TBS plus 0.1% Tween-20 for 1 h and incubated in TBS, 0.1% Tween-20, 5% NGS, and goat anti-mouse IgG (H + L) secondary antibody, Alexa Fluor™ 488 (1:500). After 3 additional washes with TBS plus 0.1% Tween-20, sections were mounted on glass slides with ProLong Gold plus DAPI (ThermoFisher, #P36931).

### ELISA

Anesthetized mice were perfused with 1 × PBS and whole brains were sagittally bisected. Right hemispheres were further dissected to separate hippocampus and cortex for downstream ELISA analyses. Hippocampal and cortical tissues were homogenized in lysis buffer (50 mM Tris, 150 mM NaCl, 1% NP40), supplemented with proteases and phosphatase inhibitors (Roche). Homogenized samples were sonicated with a probe sonicator on ice for 30 s at low level, then were centrifuged at 20,000 g for 30 min at 4 °C. Pellets obtained upon centrifugation were washed once with PBS, then resuspended in ice-cold 70% formic acid for measurement of insoluble Aβ by ELISA. Formic acid neutralization buffer (1 M Tris base, 0.5 M Na2HPO4, 0.05% NaN3) was used to adjust for pH prior to any downstream assays. Protein levels in both fractions were normalized to the protein concentration determined via Bradford protein assay. Insoluble Aβ was measured using Invitrogen Aβ42 Human ELISA Kit (#KHB3441) according to the manufacturer’s instructions. Briefly, neutralized insoluble fractions of brain tissue homogenates were loaded into precoated, flat bottom 96-well plates and to this Aβ42 detection antibody solution was added. The mixture was incubated at room temperature for 3 h with gentle shaking. The plates were then thoroughly washed with the supplied 1 × wash buffer before anti-rabbit IgG HRP was added and incubated at room temperature for 30 min. After thoroughly washing the wells with supplied 1 × wash buffer, stabilized chromogen was added to each well, following by 30-min incubation at room temperature in the dark. Stop solution was added and the absorbance was measured at 450 nm using a Cytation3 plate reader. The concentration of Aβ42 (picograms per milliliter of sample) detected in the insoluble fraction was used for statistical analysis.

### Morris water maze

MWM is a circular container (122 cm diameter; 50 cm high) filled with water (22 ± 1 °C) made opaque with non-toxic white paint. Four soft walls, each containing a visual cue consisting of four drawings with distinct shapes and bright colors, surrounded the container. Animals were acclimatized in advance to the experimentation room. Each trial was 60 s in duration. The first training trial involved placing the mouse in an enclosed rectangular channel (15 cm × 122 cm) running across the diameter of the container with a hidden platform (15 cm × 15 cm) that is 1.5 cm below the opaque water surface in the center of the channel. During subsequent training trials, no guiding channel was utilized, the hidden platform was located in a fixed quadrant, and the animal drop location was randomized. The entire pool training procedure with hidden platform and random drop location included 4 trials per day over a 4-day period, 24 h and 72 h after the last training trial, probe trials were performed in the absence of a hidden platform. In the probe trial, time spent in the platform quadrant (quadrant where the hidden platform was previously located) was measured and the number of times that the mice crossed the original platform location. Ethovision XT (Noldus Information Technology) software was used to record mice movements during trials.

### Cued fear conditioning

During the cued fear conditioning memory task, a light and tone conditioned stimulus is paired with an electric shock as unconditioned stimulus. An electric shock induces fear in the mice that is measurable as freezing behavior. The assay was performed in four computer-controlled chambers (28 × 21 × 22 cm) (Med Associates) as previously described [28]. The floor of each chamber had stainless steel rods connected to a shock scrambler and generator. During day 1 or training day, the mice were subject to 2 min of white noise of 70 dB, followed by 30 s with a light and sound tone stimulus. The last 2 s of the light and tone stimulus (conditioned stimulus) were paired with an electric shock (unconditioned stimulus) of 0.45 mA. Following, mice were subject to another 2 min of white tone, 30 s of conditioned stimulus, 2 s of conditioned and unconditioned stimulus and 2 min of white noise. During day 2 or test day, the same program of day 1 was repeated minus the unconditioned stimulus (no electric shock). This assay allowed us to assess the ability of the mice to pair the conditioned stimulus to the electric shock. Freezing behavior was recorded for all mice, and the fold change of freezing behavior during the test day (day 2) between the first conditioned stimulus time and the previous 2 min of white noise baseline was calculated.

### Elevated plus maze

The elevated plus maze consists of two open arms (without walls, 38 × 5 cm wide) and two closed arms (with walls 16 cm tall), the intersection of the arms is 5 × 5 cm wide, and the entire maze is elevated 77 cm above the ground (Hamilton-Kinder, Poway, CA). Mice were first allowed to acclimatize to the testing room for 1 h. During the test, mice were placed at the intersection between open and closed arms and were allowed to freely explore for 10 min. We assessed the exploratory behavior of the experimental mice at the elevated plus maze task by recording the time in which the center of the mouse body was located in the intersection area while the mouse dipped its head outside the open arms area.

### Y-maze

The Y-maze consisted of three 40 × 8 × 15 cm arms. Mice were first allowed to acclimatize to the testing room for 1 h. Each mouse was placed at the end of one arm, then allowed to move freely for 10 min. Total distance traveled and arm entries were monitored with the EthoVision video-tracking system.

### Cell culture

The HT-22 mouse hippocampal neuronal cell line (#SCC129, Millipore) and the SH-SY5Y human neuroblastoma cell line (#CRL-2266, ATCC) were used in this study. HT-22 cells were grown in DMEM medium, SH-SY5Y cells were grown in 1:1 DMEM/F-12. All medium was supplemented with 10% fetal bovine serum and 1% penicillin–streptomycin. All cells were grown at 37 °C and 5% CO_2_ and cultures were passaged with fresh medium two to three times per week.

### Real-time quantitative PCR for gene expression analysis

Quick-RNA MiniPrep Kit purchased from Zymo Research (#11–328) was used for total RNA extraction. The cDNA was generated according to the manufacturer’s instructions with high-capacity cDNA reverse transcription kit purchased from Thermo Fisher Scientific (#4,368,814). Primers for RT-qPCR were designed using NCBI Primer BLAST (Table S1), and RT-qPCR performed with LightCycler 480 SYBR Green I Master purchased from Roche (#04,707,516,001) using a LightCycler 480 Instrument II from Roche. The RT-qPCR reactions were performed using 10 ng/μL cDNA per reaction in technical triplicates and the fold change in gene expression was calculated with the 2(-Delta Delta C(T)) method [29], normalized to the gene expression of human β-actin (*ACTB*) or mouse glyceraldehyde-3-phosphate dehydrogenase (*Gadph*).

### DQ-BSA red

Mouse hippocampal HT22 cells were seeded in 8-well chamber slides at 15,000 cells per well. After 24 h, cells were incubated for 6 h in EBSS medium–containing 10 μg/ml of DQ-BSA red dye, supplemented with 30 μM of UA or vehicle and/or 50 μM of bafilomycin. Cells were then fixed with 4% PFA prepared in PBS for 15 min, washed three times with PBS, permeabilized for 10 min in PBS plus 0.1% Triton X-100 and mounted with ProLong Gold plus DAPI (ThermoFisher, #P36931).

### Protein extraction and immunoblotting

For total protein extraction, cells were cultured in 12-well plates and lysed in cell lysis buffer (150 mM NaCl, 1% NP-40, 50 mM Tris–HCl pH 8.0, cOmplete EDTA-free Protease Inhibitor Cocktail (#11,836,170,001) and PhosSTOP phosphatase inhibitor tablets (#PHOSS-RO) purchased from Roche), sonicated (15 cycles: 15 s on, 30 s off) with a Diagenode Bioruptor sonication system at 4 °C, and then centrifuged 20 min at 15,000 g. The supernatants were collected and boiled at 95 °C for 5 min with sample buffer (0.25 M Tris–HCl pH 6.8, 50% glycerol, 5% SDS, 0.05% bromophenol blue, and 10% 2-mercaptoethanol). The samples were then run on 4–12% gradient glycine gels (#NP0321BOX) or 16% tricine gels (#EC6695BOX) purchased from Thermo Fisher Scientific and blotted onto a 0.2 μm PVDF membrane purchased from Millipore (#ISEQ00010). The membranes were blocked with 3% BSA in PBS-T (0.1% Tween 20 in PBS) for 1 h at room temperature, and then stained overnight at 4 °C with 3% BSA in PBS-T and the primary antibodies referenced above. Membranes were washed with PBS-T and stained for 1 h at room temperature with fluorescent secondary antibodies: anti-mouse IgG(H + L) DyLight 800 conjugate (#5257, CST) and anti-rabbit IgG(H + L) DyLight 680 conjugate (#5366, CST). The images were captured using a LiCor Odyssey Infrared Imaging System 9120 (LI-COR Biosciences).

### Statistical analysis

Confocal microscopy data is presented as mean ± SEM; *p* value was calculated via unpaired *t* test. MWM, cued fear conditioning, elevated plus maze and y-maze data are presented as mean ± SEM; *p* value was calculated via two-way and one-way ANOVA. Survival plot data *p* value was calculated via Gehan-Breslow and Log-rank tests. Analyses of qPCR, DQ-BSA red fluorescence, and Western blot are presented as mean ± SEM; *p* value was calculated via unpaired *t* test. Graphpad Prism 7 was used for all the statistical analyses. Artwork was created using ImageJ, Graphpad Prism 7 and Illustrator.

## Results

### Urolithin A reduces Aβ burden in 3xTg-AD mice

**Fig. 1:**
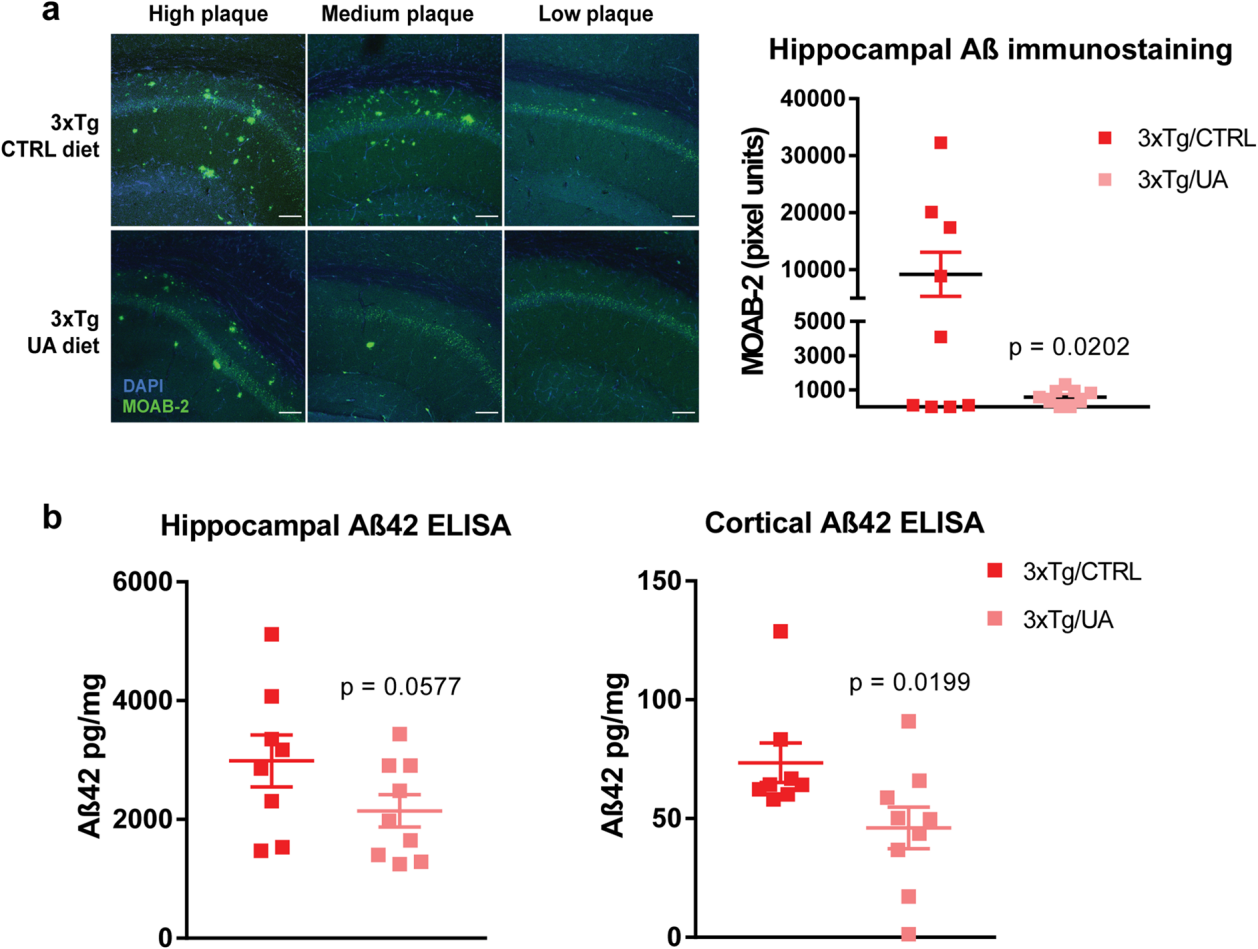
Urolithin A (UA) reduces Aβ burden in 3xTg-AD mice. **A** Confocal microscopy images of Aβ42 + Aβ40 immunostaining (detected with the MOAB-2 antibody) in the rostral hippocampus of 14-month-old female mice (*n* = 9). Images depict representative inter-subject variability with high, medium and low Aβ content in each experimental group. Scale bars = 100 μm. Corresponding pixel quantification data is presented as mean ± *SEM*; *p*-value was calculated via unpaired t-test. (**B**) ELISA immunodetection of insoluble Aβ42 in 14-month-old female mice (*n* = 8–9). NP40-insoluble hippocampal and cortical fractions were solubilized in 70% formic acid (FA) fractions. Data is presented as mean ± *SEM*; *p*-value was calculated via unpaired *t* test

Previous studies have demonstrated that UA can act as a potent inducer of mitophagy [19, 20, 22], capable of ameliorating dysfunctional mitochondrial function in both an Aβ42 *Caenorhabditis* (*C.*) *elegans* model and the APP/PS1 mouse model of AD [21, 30]. Here, we explored the effects of UA dietary supplementation on the age-dependent progression of Aβ pathology in 3xTg-AD mice. 3xTg-AD mice are a useful model of late-onset AD (LOAD) as they display several cardinal phenotypes of the human disease including age-related accumulation of Aβ and phosphorylated Tau species, gliosis, and eventual neuronal loss, accompanied by progressive cognitive deficits [27, 31]. Female mice were used for these as they have been reported to display earlier and more consistent Aβ pathology than that observed in males [32]. Immunocytochemistry was performed using an Aβ-human-specific monoclonal antibody (MOAB-2, *n* = 9 per experimental group). Hippocampal sections from left hemibrains were − 2.2 mm caudal to bregma, representing sections of the rostral hippocampus. Extracellular plaques were detected in the CA1 and subicular regions of the rostral hippocampus in 14 out of 18 3xTg-AD animals. In contrast, no immunoreactive plaques were found in age-matched wild-type mice (Fig. S1a). Quantitative analysis of Aβ immunoreactivity in sections obtained from 3xTg-AD animals revealed that overall plaque burden was reduced in mice fed the UA-supplemented diet (unpaired *t* test analysis, *p* value = 0.0202; Fig. 1a).

We performed additional Aβ42 quantification in hippocampal and cortical homogenates via sandwich ELISA using an antibody specific for this species (6E10). Unpaired *t* test analysis revealed a trend toward decreased hippocampal Aβ in UA-fed animals (unpaired *t* test analysis, *p* value = 0.0577; Fig. 1b) and a statistically significant decrease in cortical Aβ in UA-fed animals (unpaired *t* test analysis, *p* value = 0.0199; Fig. 1b).

We next proceeded to measure phospho-tau immunoreactivity in these same sections. For this purpose, we utilized the AT8 antibody specific to phosphorylated Ser202 and Thr205 residues. Whereas no immunoreactivity was detected in the sections extracted from wild-type mice (Fig. S1a), specific staining pertaining to phospho-tau was observed in a number but not all 3xTg-AD mice (Fig. S1b). Due to the inconsistency in phospho-tau immunoreactivity across the experimental subjects examined, we were not able to determine the statistical impact of UA on this marker of tau pathology at this time point.

### *Urolithin A prevents spatial learning and memory deficits in 3xTg-AD* mice

**Fig. 2:**
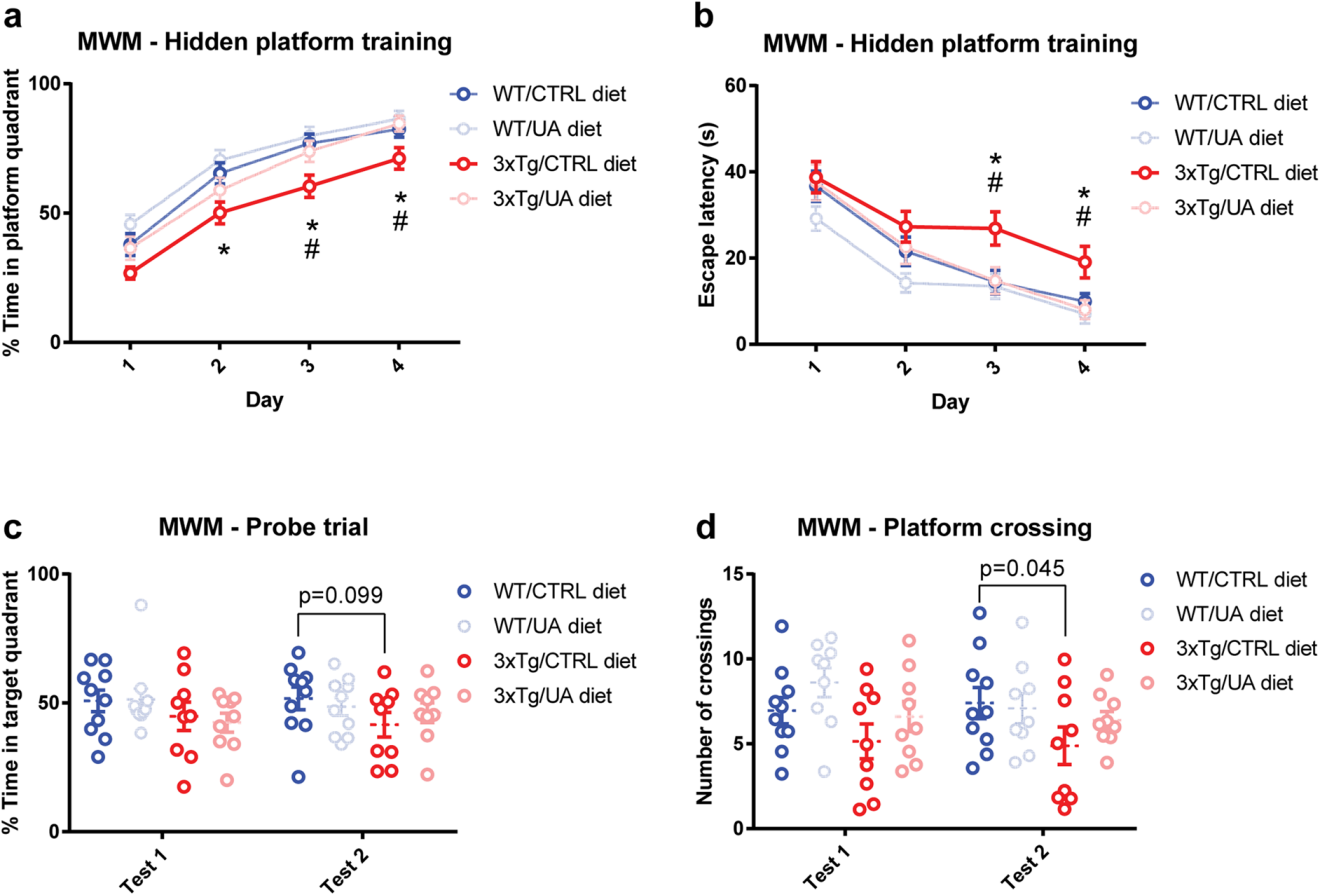
UA prevents spatial learning and memory deficits in 3xTg-AD mice. Different parameters measured during the trial and probe phases of the Morris Water Maze (MWM): **A** Percent of time spent in platform quadrant; **B** escape latency; **C** percent of time in target quadrant during probe test and **D** number of platform crossings during the probe test normalized by swim velocity. All values from 13-month-old female mice (*n* = 9–10) were quantified as mean ± *SEM*; *p* value was calculated via two-way ANOVA with pairwise post hoc Fisher LSD test; * WT/CTRL vs. 3xTg/CTRL *p* value < 0.05. ^#^ 3xTg/CTRL vs. 3xTg/UA *p* value < 0.05

Previous studies have demonstrated that UA is neuroprotective and can rescue cognitive deficits in the APP/PS1 mouse model [21, 30]. We evaluated spatial memory using the well-established Morris water maze (MWM) [33, 34] in 13-month-old female 3xTg-AD mice. The test involves a 4-day learning period followed by two probe trials 24 h and 72 h after the last learning session. During each learning trial, time spent in the platform quadrant and escape latency were recorded (the latter defined as time needed to find the platform). During the probe trials, time spent in the platform quadrant and the number of platform crossings were recorded (the latter defined as number of instances of swimming over the area where the platform used to be in the previous learning trials). 3xTg-AD mice (3xTg/CTRL) spent less time than wild-type mice (WT/CTRL) in the platform quadrant during the training period (Fig. 2a) and required a significantly longer time to find the platform (Fig. 2b). In a manner similar to wild type, UA-fed 3xTg-AD mice (3xTg/UA) spent significantly more time in the platform quadrant (Fig. 2a) and required significantly less time to find the platform than the 3xTg/CTRL (Fig. 2b). During the first probe trial, no group among the four experimental groups (WT/CTRL, WT/UA, 3xTg/CTRL, and 3xTg/UA) showed any significant difference in the time spent in the target quadrant (where the platform formally resided) (Fig. 2c). In the second probe trial, there was a trend (*p* value = 0.099) for the 3xTg/CTRL to spend less time in the target quadrant as compared to the WT/CTRL (Fig. 2c). Similarly, the number of platform crossings for 3xTg/CTRL did not significantly differ from the other experimental groups during the first probe trial (Fig. 2d), but during the second probe trial 3xTg/CTRL crossed the target a significantly reduced number of times compared to WT/CTRL (Fig. 2d). Importantly, 3xTg/UA mice did not demonstrate a similar significant decrease in the number of platform ﻿crossings during the second probe trial, suggesting that UA feeding improved spatial learning of the 3xTg-AD mice. To summarize these findings, 13-month-old 3xTg-AD mice display significant deficits in spatial learning and memory that are successfully prevented by UA diet supplementation, suggesting a positive effect on cognition, likely linked to the observed reduction in neuropathology (Fig. 1).

### Urolithin A ameliorates associative learning and exploratory behavior in 3xTg-AD mice

**Fig. 3:**
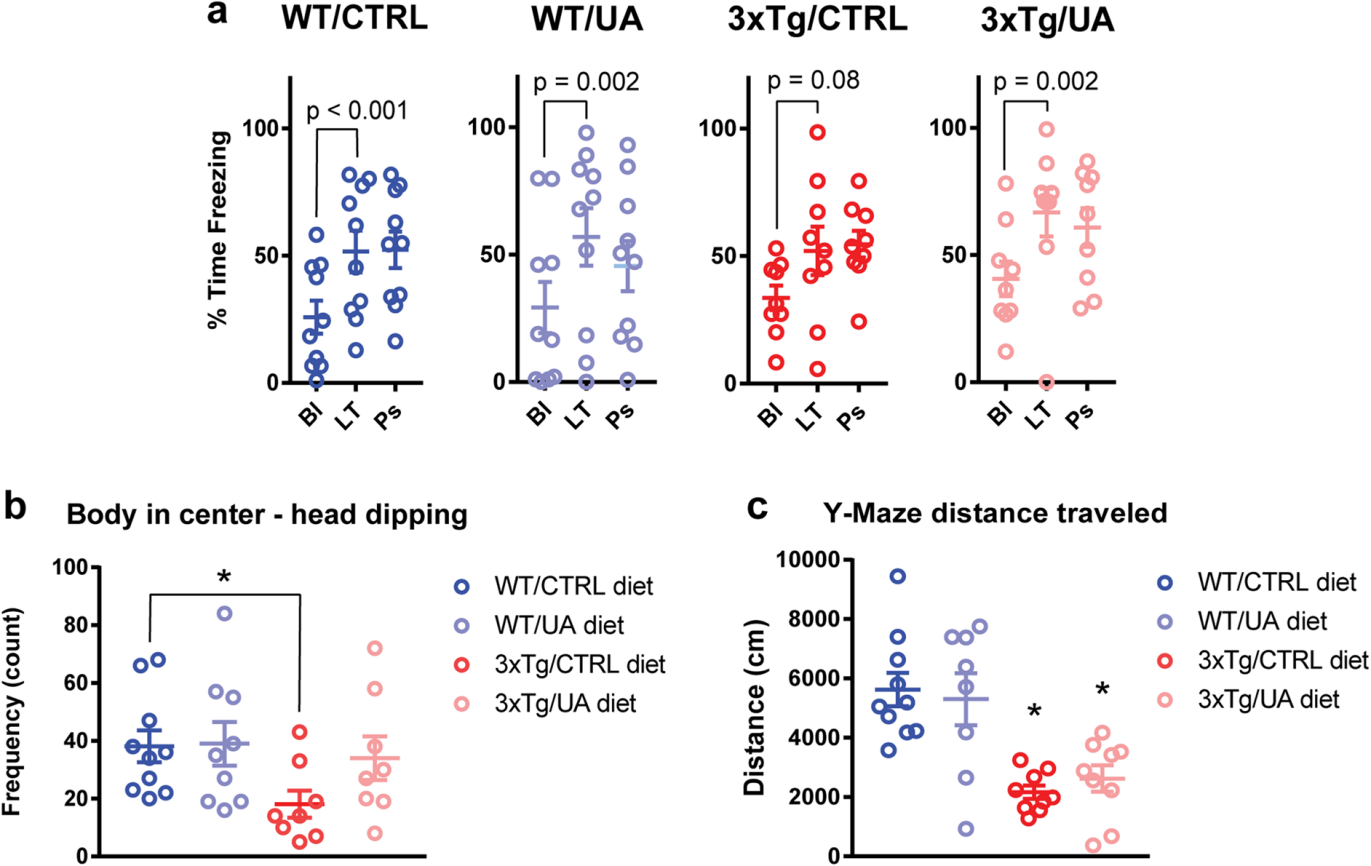
UA ameliorates deficits in associative learning and exploratory behavior in 3xTg-AD mice. **A** Cued fear conditioning test: percentage of time spent frozen, where Bl refers to their baseline freezing, LT refers as freezing during application of light and tone cues and Ps refers to freezing after the cues stopped. Data was quantified as mean ± *SEM* (*n* = 9–10) in 12-month-old female mice. One-way repeated measures ANOVA with pairwise post hoc Fisher LSD test. **B** Elevated plus maze: exploratory behavior measured as count of head dip movements while animal body was located at maze center. Here decreased head dipping in the 3xTg/CTRL mice, indicate a depression in exploratory behavior, which is reversed in the 3xTg/UA mice. Data was quantified as mean ± SEM (*n* = 9–10) in 12-month-old female mice. *One-way ANOVA with pairwise post hoc Fisher LSD test *p* value < 0.05. **C** Y-maze: total distance traveled as a measure of baseline exploratory behavior, which is reduced in the 3xTg-AD mice. Data was quantified as mean ± SEM (*n* = 9–10) 12-month-old female mice. *One-way ANOVA with pairwise post hoc Fisher LSD test *p* value < 0.05

In order to assess associative learning ability in 3xTg-AD mice, we performed a cued fear learning task, whereby mice learn to associate visual and auditory cues with an electric shock and display a freezing behavior upon presentation of the cues [35]. 12-month-old WT/CTRL, WT/UA, and 3xTg/UA displayed significantly increased freezing behavior upon cues (Fig. 3a). In contrast, 3xTg/CTRL increased their freezing behavior upon these cues to a lesser extent, not statistically significant, indicative of deficits in associative learning (Fig. 3a).

We used the elevated plus maze to measure the number of times a mouse located in the center of the maze would dip its head below the level of the maze in the open arm, a behavior that can indicate willingness to explore the surroundings [36]. We found that the 12-month-old 3xTg/CTRL performed significantly fewer head dips than age-matched WT/CTRL and 3xTg/UA (Fig. 3b).

Locomotor activity and willingness to explore were also measured using the Y-maze task [37]. 12-month-old 3xTg-AD mice, regardless of diet, displayed reduced locomotion and, reduced exploratory behavior in the Y-maze (Fig. 3c).

Additionally, we used the Y-maze to assess whether the 3xTg-AD mice displayed any deficits in working memory as measured by the percentage of spontaneous alternations amongst all the arm alternation triads performed by each mouse. We did not observe a statistically significant change in the spontaneous alternation behavior percentage across any experimental groups (Fig. S2a). In contrast, we measured a significantly reduced number of arm entries in both 3xTg/CTRL and 3xTg/UA (Fig. S2b). Arising from the observation that a mouse may explore the same arm back and forth without making additional arm entries, we decided to analyze the number of spontaneous alternations triads normalized by distance traveled. Here, we observed that there is a trend toward a decrease in the number of spontaneous alternations performed per distance traveled in the 3xTg/CTRL, which is less pronounced in the 3xTg/UA (Fig. S2c). Significance was likely reduced by a large degree in variability in behavior within each experimental group and the marked reduction in locomotor activity displayed by the 3xTg-AD mice.

### Hippocampal Aβ burden positively correlates with spatial learning and memory in control-fed but not in UA-fed 3xTg-AD mice

**Fig. 4:**
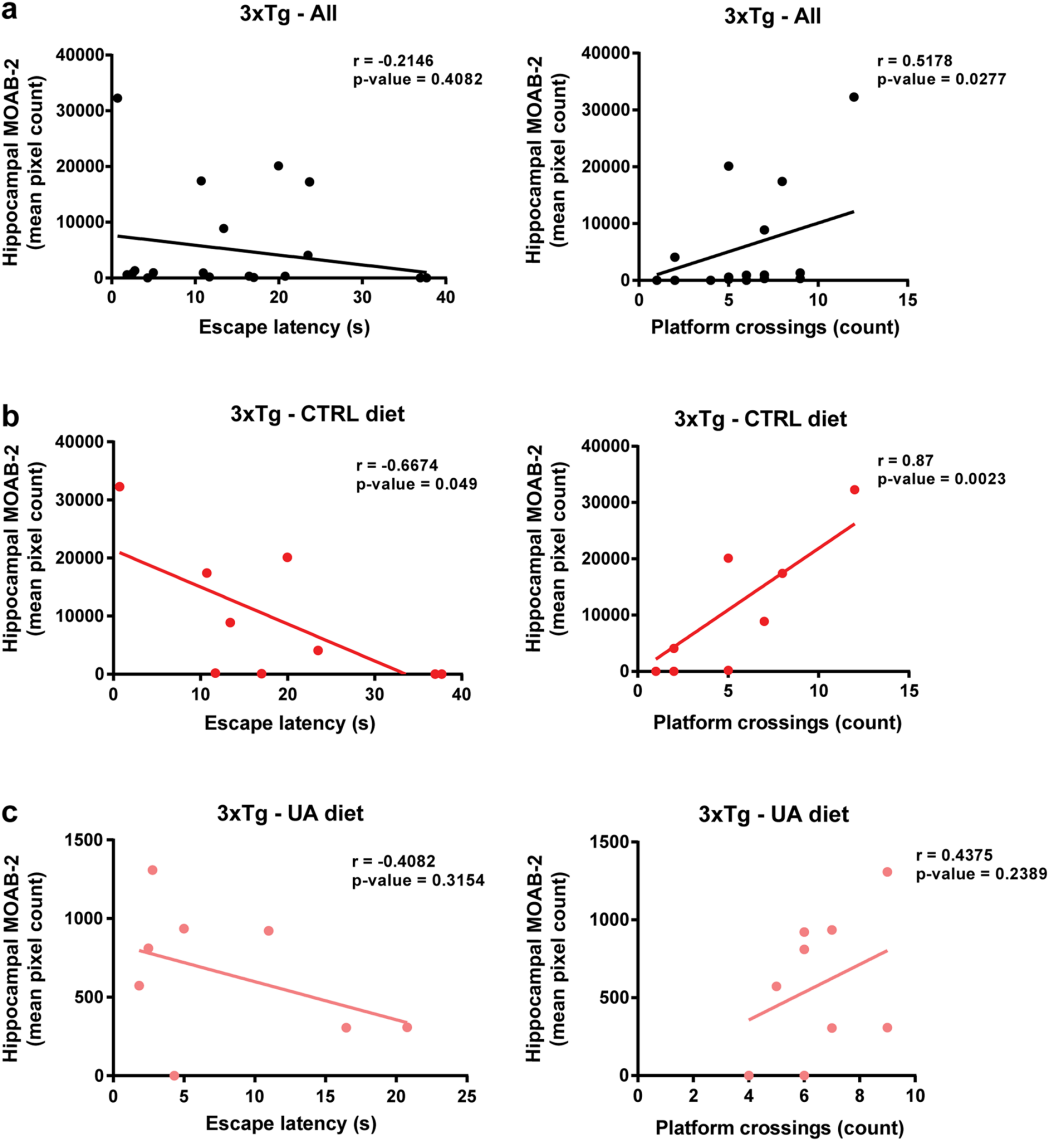
Hippocampal Aβ-containing plaques positively correlate with spatial learning and memory in 3xTg-AD mice. Analysis of the linear correlation between the extension of Aβ plaques in the hippocampus of mice (as depicted in Fig. 1A) vs. spatial learning and memory assessed in the MWM. Aβ plaques vs. escape latency- day 4 (graphs on right) or Aβ plaques vs. platform crossings- second probe test (graphs on left). Data is shown for **A** all 3xTg-AD subjects, regardless of diet (*n* = 18); **B** 3xTg-AD mice on control diet (*n* = 9), **C** 3xTg-AD mice on UA diet (*n* = 9)

Aberrant accumulation of Aβ is a cardinal sign of AD and has been robustly associated with neuropathology and cognitive deficits in humans and in animal models. As previously noted, UA diet supplementation resulted in decreased hippocampal Aβ burden in the 3xTg-AD mice and significantly prevented cognitive deficits associated with this mouse model. Based on these observations, we analyzed whether hippocampal Aβ protein levels impacted spatial learning and memory performance in the MWM at the level of the individual animal. When we plotted the hippocampal Aβ-immunoreactive pixel count in 3xTg-AD mice against both the escape latency and the number of platform crossings, we found that while Aβ protein levels did not correlate with escape latency, they were positively correlated with the number of platform crossings (*r* = 0.5178, *p* value = 0.0277) (Fig. 4a). Next, we performed correlation analyses between Aβ42 abundance in triton-insoluble/formic acid-soluble cortical and hippocampal homogenates and escape latency and, similarly, no correlation was found between Aβ42 abundance in cortical or hippocampal homogenates and escape latency in all 3xTg-AD subjects (Fig. S3a). We then further dissected these analyses, splitting the two 3xTg-AD experimental groups according to their diet. Interestingly, there was a strong negative correlation between hippocampal Aβ plaque burden and escape latency (*r* =  − 0.6674, *p* value = 0.049) and a strong positive correlation between hippocampal Aβ burden and number of platform crossings (*r* = 0.87, *p* value = 0.0023) in the 3xTg/CTRL (Fig. 4b). In line with this, a trend toward a negative correlation was found between escape latency and Aβ42 in the hippocampus homogenates of the 3xTg/CTRL subjects (*r* =  − 0,693, *p* value = 0.0567) (Fig. S3b). In contrast, in the 3xTg/UA mice, the correlation between hippocampal Aβ plaque burden and escape latency or number of platform crossings were weaker and not statistically significant (Fig. 4c). Additionally, a significant positive correlation was observed between escape latency and Aβ42 in the cortical (*r* = 0.6887, *p* value = 0.0489) and hippocampal homogenates (*r* = 0.7849, *p* value = 0.021) of the 3xTg/UA mice (Fig. S3c).

To summarize our findings on the correlation between Aβ burden and spatial learning and memory, we found that in the control diet-fed 3xTg-AD mice (Fig. 4b), higher amount of Aβ extracellular plaques in the hippocampus was associated with more intact spatial learning and memory. In contrast, in 3xTg/UA mice (where plaque burden is significantly lower than it is in 3xTg/CTRL mice) both cortical and hippocampal Aβ42 abundance correlated with reduced spatial learning and memory. These findings suggest that extraneuronal Aβ plaque formation may be a protective mechanism that mitigates pathology associated with accumulation of Aβ, which if allowed to accumulate would otherwise perturb spatial learning and memory.

### UA extends median survival in male C57BL/6 mice

**Fig. 5:**
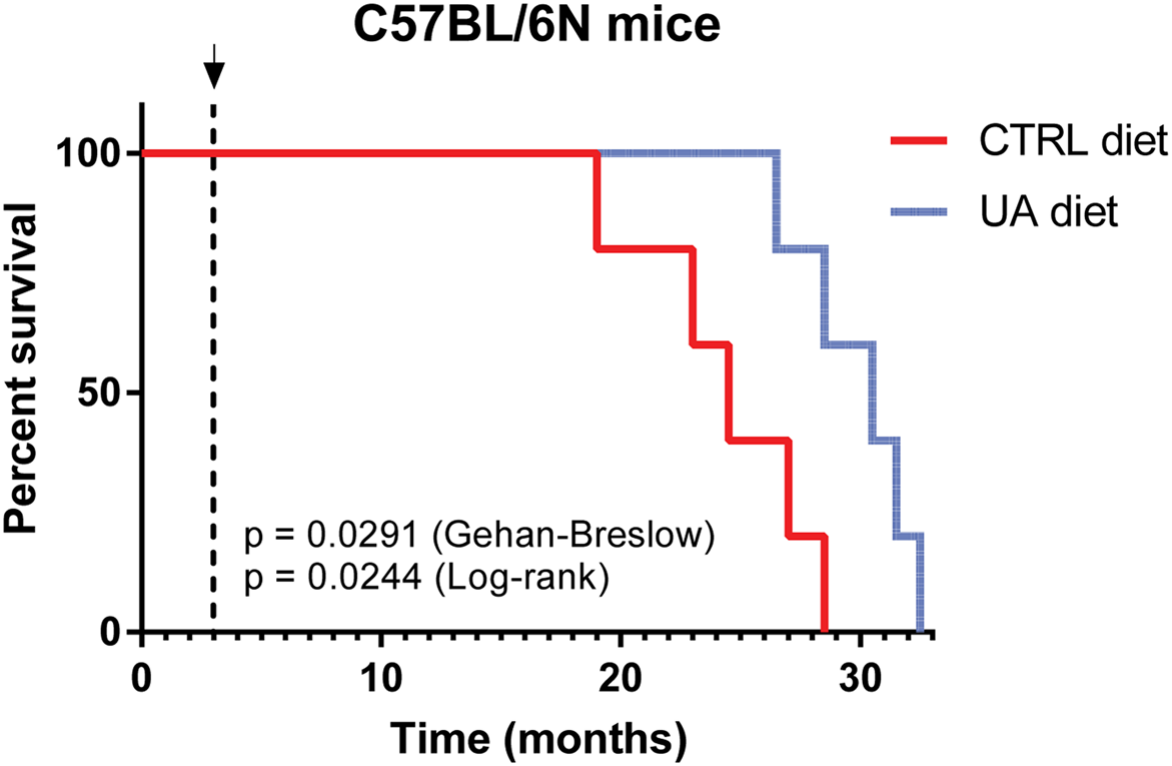
UA significantly extends lifespan in C57BL/6NJ mice. Survival plot of male mice (*n* = 5). Mice were fed regular chow or chow supplemented with 25 mg/kg of UA in alternate weeks (1 week on, 1 week off) starting at 3 months of age as indicated by the black arrow and dashed line

Mitophagy and autophagy are cellular processes known to be implicated in aging and lifespan [38, 39]. Disruptions in this cellular process have been associated with reduced lifespan across various species, including vertebrates [40–43]. Following our observations that UA can reduce Aβ abundance in the brain of 3xTg-AD mice and reverse associated cognitive deficits, we investigated whether long-term intermittent administration of the compound would be able to enhance lifespan in normal aging mice. For this purpose, we fed UA on alternate weeks (1 week on, 1 week off) to a cohort of male C57BL/6 mice starting at 3 months of age. We found that dietary supplementation of UA significantly increased survival (Fig. 5). The median lifespan at 80th percentile mortality was extended 18.75% from the UA feeding start point (Log-rank test: chi square = 5.068, *p* value = 0.0244).

### UA induces the expression of autophagy-related genes, increases autophagy flux and clears Aβ in neuronal cells in vitro

**Fig. 6:**
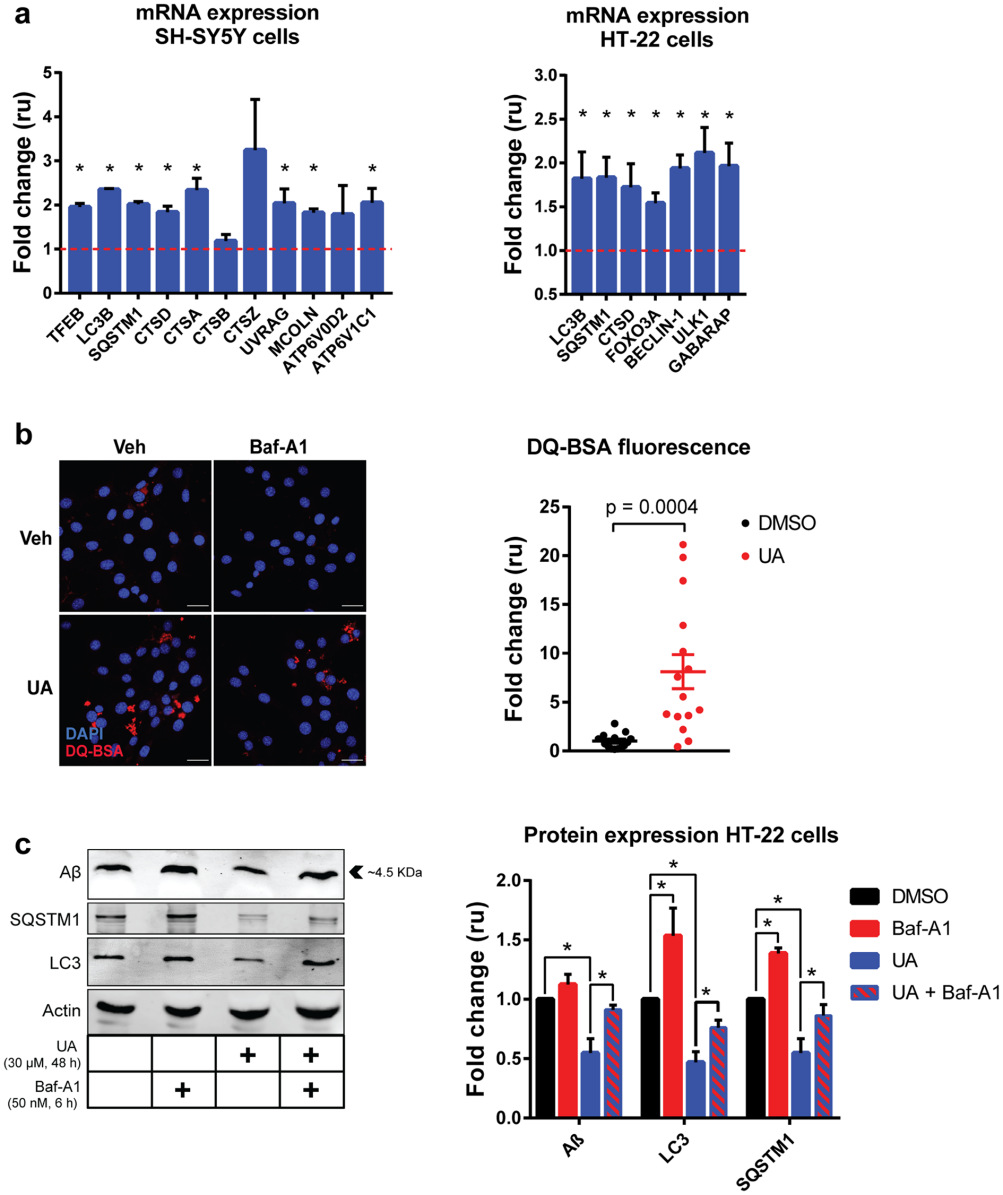
UA induces the expression of autophagy-related genes, increases autophagy flux and clears Aβ in vitro*.* **A** Relative expression of autophagy-related genes assessed via qPCR from mRNA extracted from SH-SY55 (human neuroblastoma—right side) and HT-22 (mouse hippocampal—left side) cells treated with 30 μM UA versus vehicle-treated controls (DMSO) for 24 h. Data is presented as mean fold change in relative units (ru) ± SEM by normalizing expression of each gene to that of human β-actin (*ACTB*) or mouse glyceraldehyde-3-phosphate dehydrogenase (*Gapdh*). *p value < 0.05 calculated via unpaired *t* test. **B** Representative single-plane confocal micrographs of HT-22 cells treated with or without UA (30 μM) and/or bafilomycin (50 nM) showing fluorescence of DQ-BSA red (red signal) and DAPI nuclear staining (blue signal) after 6 h of uptake and respective drug treatments. Scale bars = 30 μm. Corresponding pixel quantification data is presented as mean fold change in relative units (ru) ± *SEM*; *p* value was calculated via unpaired *t* test, *n* = 3. **C** Western blot analysis of amyloid beta, SQSTM1, and LC3B protein levels, using specific antibodies in HT-22 cells treated with Aβ oligomers (2 μM, 48 h), with or without UA and/or bafilomycin. Shown is a representative figure for four independent experiments. Quantification of changes in protein expression is presented as mean fold change in relative units (ru) ± SEM by normalizing each protein’s band intensity to the expression of actin and are presented as a fold-change relative to the samples treated with DMSO as vehicle. *p value < 0.05 calculated via unpaired *t* test

Increased expression of mitophagy-related genes and proteins has been observed following UA administration in both *C. elegans* and in mouse muscle cells and tissues [20, 26]. Given the ability of UA to mitigate neuropathology in the 3xTg-AD mouse model, we looked at the impact of UA in more tractable neuronal cell culture models to determine whether its effects could be elicited by enhanced macroautophagy and subsequent removal of Aβ. For this purpose, we utilized mouse hippocampal cells and SH-SY5Y human neuroblastoma cells in order to determine whether UA can modulate endosomal biogenesis and the autophagy pathway. We performed gene expression and western blot studies and found that UA induces the expression of a panel of autophagy-related genes in both murine and human neuronal cell types (Fig. 6a). Western blot analysis of human neuroblastoma cell lysates showed that 30 μM UA for 24 h caused a marked decrease in protein levels of sequestosome-1 (SQSTM1) and microtubule-associated protein light chain 3 (LC3) upon UA treatment (Fig. S4). Decreased expression of both proteins is a well-established readout of autophagy flux, indicating their effective clearance by degradative autophagosomes [44]. Next, we assessed whether UA’s role at inducing autophagy-related gene expression and autophagosomal degradation was preceded by increased endosomal acidification suggestive of enhanced autophagic capacity [45, 46]. Using DQ-BSA self-quenching staining which emits bright fluorescence upon its fragmentation in functional acidic endosomes [47], we found that UA significantly increased DQ-BSA fluorescence in mouse hippocampal cells after 6 h of treatment (Fig. 6b).

Following the observations that UA can enhance the turnover of the endolysosomal pathway and autophagy, we decided to perform an in vitro assessment of UA’s ability to augment the clearance of exogenous Aβ oligomers. We treated mouse hippocampal cells with Aβ oligomers for 48 h in the presence or absence of UA co-treatment. In cells treated with UA, Aβ is less abundant after 48 h, suggesting that UA’s ability to upregulate autophagy leads to improved clearance of the exogenous Aβ species (Fig. 6c). Simultaneous treatment of mouse hippocampal cells with UA and an inhibitor of autophagy, bafilomycin, partially reverses the clearance of Aβ. In parallel, levels of SQSTM1 and LC3 proteins, as a read-out of autophagic flux, were found to be decreased in cells treated with UA. In contrast, co-treatment with bafilomycin to block autophagosomal acidification and degradation reversed the depletion of both autophagy-related proteins (Fig. 6c).

Collectively, these data suggest that UA can effectively increase autophagic function in neuronal cell types in a mechanism that involves the upregulation of autophagy-related gene expression, increased numbers of acidic endosomes and enhanced clearance of autophagosomes as measured by a marked decrease in the protein levels of LC3 and SQSTM1. Additionally, exogenous addition of Aβ oligomers to mouse hippocampal cells leads to detection of Aβ proteins in the lysates. Aβ abundance decreased following UA treatment in an autophagy-dependent manner, as pharmacologically inhibiting autophagosomal acidification led to abrogation of UA-mediated Aβ clearance.

## Discussion

Biomedical research is increasingly demonstrating an association between defective lysosomal function and various pathological processes in humans, including inherited lysosomal storage disorders [48], cancer [49–51], and neurodegeneration [52–54]. Autophagic dysfunction is a commonly observed pathological feature in neurodegeneration associated with both transgenic animal models and human AD patients [55–58]. In AD, accumulation of lysosomes and subsequent blockage of lysosomal function has been described as a cause for disrupted axonal vesicle transport and axonal dystrophy [59]. Therefore, modulation of the autophagic process and the development of therapeutics to that effect are of general interest, not only in AD, but also other neurodegenerative disorders including Huntington’s and Parkinson’s disease [60, 61].

UA is a benzo-coumarin produced by the human gut microbiota. It has been gathering significant attention due to its mitophagy-inducing properties, which are thought to drive its beneficial health effects in muscle and in the central nervous system across several species and disease models. In our study, we utilized the 3xTg-AD mouse model to uncover potential therapeutic benefits of UA in the context of AD-associated pathology. The 3xTg-AD mouse model is highly regarded as a late onset Alzheimer’s disease (LOAD) model, as it displays progressive neuropathology and behavioral and cognitive impairment with age [27, 60]. The 3xTg-AD model displays sex differences, with increased Aβ levels and more extensive cognitive deficits being reported in female mice [31, 32]. A sex-bias featuring enhanced pathology in females has also been observed in another commonly used mouse model of AD, the 5xFAD-AD model. AD is also found to be more prevalent in women. For this reason, we chose to conduct our study using female mice only. Importantly, genetic drift has been documented in the 3xTg-AD model such that, compared to original findings, there is now a delay in the presentation of its neuropathological hallmarks and associated neurodegeneration, and this delay is particularly pronounced in males [31, 61]. This is likely the reason that while we were able to detect extensive amyloidosis at 13 months of age in this model, particularly in the hippocampus, we did not detect the same level of tauopathy at this same time point. Nonetheless, deficits in spatial learning, associative learning, exploratory behavior, and locomotion were apparent at this pathological stage.

Following administration of UA starting at 3 months of age for a period of 10 months, we observed a significant reduction in Aβ-positive plaque load in the hippocampus and in Aβ42 in cortical homogenates, as well as a trend toward a reduction of Aβ42 in hippocampal homogenates. Our findings are in line with a previous study that showed a decrease in Aβ plaque formation in the hippocampus of APP/PS1 mice following UA administration [21]. In 13-month-old female 3xTg-AD mice, Aβ40/42 positive plaques were predominant in the hippocampus and absent from the neocortex, whereas Aβ42 concentration in hippocampus homogenates was 40–50 × more abundant than in the neocortex. In order to explain the differences, we observed between reductions in Aβ-positive plaques, and Aβ42 in hippocampal homogenates, we took into account differences in expression of Aβ40 and Aβ42 and whether these proteins were localized intracellularly or present in the dense extracellular plaques. We found high variability of Aβ burden across subjects suggesting that within the same 3xTg-AD genotype, some animals display high Aβ burden and others have significantly lower cerebral Aβ expression. Within the UA diet experimental group, there may additionally be variability in response to UA treatment. We determined that while behavioral performance is highly variable at the 12–13-month time point between individual animals, overall 3xTg-AD mice display age-related deficits in spatial learning and memory, associative learning and exploratory behavior that were prevented by UA diet supplementation.

Intriguingly, our correlation analyses showed that hippocampal Aβ plaque load positively correlated with superior spatial learning and memory in individual 3xTg-AD mice fed normal diet, but not in those on the UA diet. Increased Aβ immunoreactivity and extensive plaque formation has also been found in clinically healthy patients [62–64], whereas neuronal loss poorly correlated with plaque formation in AD patients [65, 66]. As discussed by other groups, confinement of Aβ deposition within dense confined regions of the hippocampus may be a key protective mechanism that blunts intracellular Aβ-mediated neurotoxicity and neurodegeneration [67–69]. This hypothesis, and the observations that support it, should bring cautious scrutiny to study designs that aim to disrupt the formation and/or solubilize dense-core Aβ plaques, as such aims may ultimately prove ineffective or even hazardous in mitigating AD symptomology.

UA and other therapeutic agents that prove to be potent inducers of the autophagic machinery could enhance clearance of intracellular Aβ and/or boost the action of phagocytic microglia to remove smaller extracellular Aβ deposits, before the formation of dense-core plaque is required to control further neurological damage [70]. Using cortical and hippocampal homogenates, we found that in UA-treated 3xTg-AD mice (whose hippocampi show less dense-core plaque pathology than 3xTg-AD mice on regular diet), both cortical and hippocampal Aβ42 levels correlated with poorer spatial learning and memory. In our view, this indicates that Aβ42 abundance outside of dense-core extracellular plaques might be a superior indicator of neuropathology and cognitive decline than quantification of plaque load.

We further demonstrated that UA is an effective inducer of macroautophagy in two separate neuronal cell lines. UA increased the expression of a panel of autophagy-related genes in both cell types and was effective in reducing the protein levels of LC3 and SQSTM1, two well-established readouts of autophagy that are acutely depleted upon increased autophagosomal degradation [44]. In Western blot analyses, LC3 resolves as a doublet of the upper band LC3-I, and the lower band LC3-II. When the autophagic process is initiated, a phosphatidylethanolamine group is added to LC3-I, causing its conversion to LC3-II [71]. Of note, in human neuroblastoma cells, the expression of non-phospholipidated LC3-I is high and UA treatment enhances its conversion to LC3-II in tandem with a reduction in overall LC3 levels. In contrast, in mouse hippocampal cells, lipidated LC3-II is predominant and UA treatment significantly reduces its protein levels indicating increased autophagosomal clearance.

In addition, UA increased the abundance of autophagy-functional acidic endosomes. This is in line with previous reports that described augmented autophagy-related gene expression in UA-treated worms and muscle tissue. Our results further show that in mouse hippocampal cells treated with Aβ oligomers, UA co-treatment led to a decrease in Aβ protein levels in whole cell lysates. UA-mediated Aβ clearance was found to be dependent on autophagy, as clearance was blunted by treating the cells with bafilomycin.

Finally, in terms of its efficacy as an anti-aging agent, UA has previously been demonstrated to extend lifespan in the invertebrate nematode *C. elegans* [20]. In our study, we show evolutionary conservation of this anti-aging property as significant lifespan extension was observed in normal aging mice intermittently fed UA. Follow-up studies that increase the sample size and include both female and male subjects are warranted to assess the efficacy of UA, as a potential rejuvenating agent.

Our findings in the 3xTg-AD mouse model importantly suggest that dense extracellular Aβ plaques are not necessarily predictive of cognitive decline. Our results may speak to the ongoing and highly publicized controversies surrounding AD clinical trials, where therapeutics designed to expressly prevent plaque formation, while often efficacious in lessening plaque load, are routinely and notoriously ineffective at meeting their primary endpoint, namely preventing of slowing cognitive decline [72, 73]. We show that UA is an inducer of macroautophagy that may be useful in reducing Aβ build-up and that long-term intermittent autophagy enhancement can extend lifespan in rodents. Lysosomal dysfunction is increasingly found at the core of neurodegenerative disorders where proteotoxicity manifests and restoration of the autophagic pathways may prove to be a successful therapeutic approach at slowing the progression of AD and other age-related disorders.

## Supplementary information

Below is the link to the electronic supplementary material.

Supplementary file1 (DOCX 2445 KB)

## Data Availability

The data that support the findings of this study are available from the corresponding authors (JB, JKA) upon request.

## Abbreviations

AD: Alzheimer’s disease
UA: Urolithin A
Aβ: Amyloid beta
APP: Amyloid precursor protein
PSEN: Presenilin
*C.*: *Caenorhabditis*
LOAD: Late-onset AD
*SEM*: Standard error of the mean
FA: Formic acid
MWM: Morris water maze
Bl: Baseline
LT: Light and tone
Ps: Post-shock
SQSTM1: Sequestosome-1
LC3: Microtubule-associated protein light chain 3
DMSO: Dimethyl sulfoxide
ru: Relative units
*ACTB*: β-Actin
*Gadph*: Glyceraldehyde-3-phosphate dehydrogenase
IACUC: Institutional Animal Care and Use Committee
