# Supplementary material for: Urolithin A reduces amyloid-beta load and improves cognitive deficits uncorrelated with plaque burden in a mouse model of Alzheimer’s disease
Source: GeroScience. 2022 Dec 28;45(2):1095–113. doi: 10.1007/s11357-022-00708-y (PMC9886708; doi:10.1007/s11357-022-00708-y)
Supplement: Supplementary file 1 — Supplementary file1 (DOCX 2445 KB) [file 11357_2022_708_MOESM1_ESM.docx]

Urolithin A reduces amyloid-beta load and improves cognitive deficits uncorrelated with plaque burden in a mouse model of Alzheimer’s disease

GeroScience

Josué Ballesteros-Álvarez^1^, Wynnie Nguyen^1^, Renuka Sivapatham^1^, Anand Rane^1^ and Julie K Andersen^1*^

*Corresponding author: Julie K Andersen (JKA); [jandersen@buckinstitute.org](mailto:jandersen@buckinstitute.org), (415)-717-9761

**
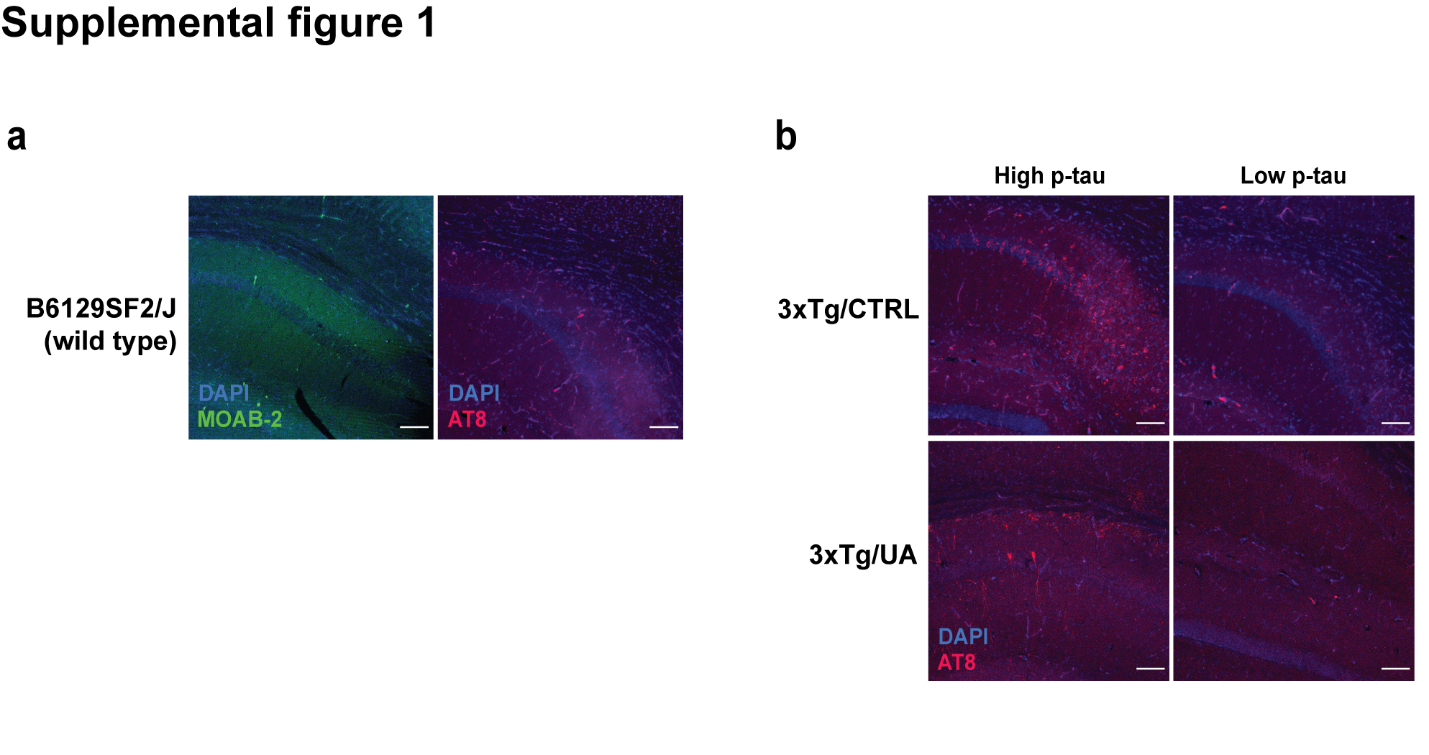
Fig. S1** Aß is undetectable in the rostral hippocampus B6129SF2/J (wild type) mice whereas detection of phosphorylated tau protein at Ser202/Thr205 suggests initial development of tauopathy in 14-month-old female 3xTg-AD mice. (A) Confocal microscopy images showing *absence* of Aß42+Aß40 (MOAB-2; green; left) and of phosphorylated tau at Ser202/Thr205 (AT8; red; right) immunostaining in the rostral hippocampus of 14-month-old female wild type mice (*n* = 10). Scale bars = 100 µm. (B) Confocal microscopy images showing phosphorylated tau protein at Ser202/Thr205 in the rostral hippocampus of 14-month-old female 3xTg-AD mice. Images depict representative inter-subject variability with high and low phospho-tau content in each experimental group. Scale bars = 100 µm

**
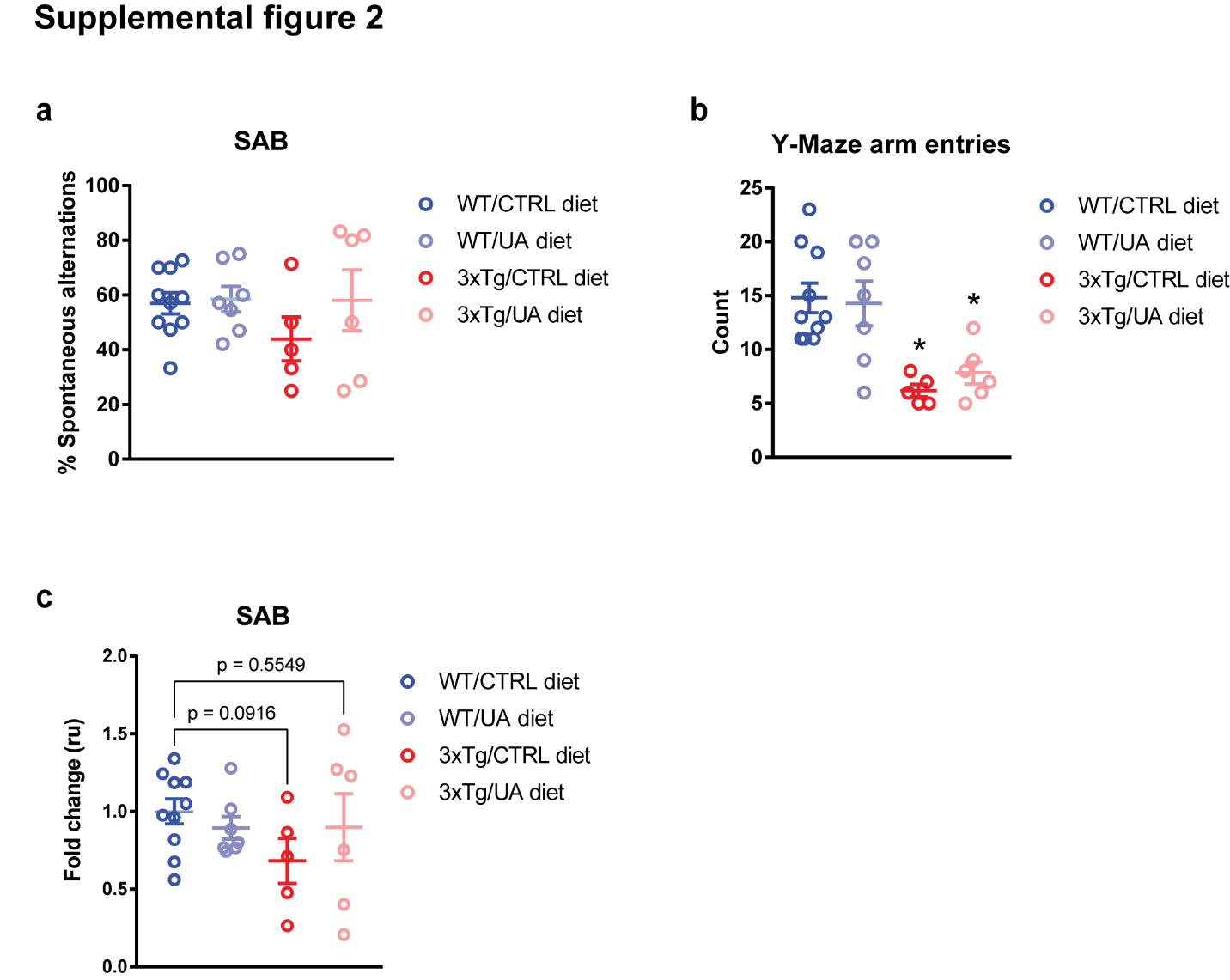
**

**Fig. S2** 3xTg-AD mice display a significant reduction in locomotor activity and perform fewer entries in the Y-Maze task. (A) Spontaneous alternation behavior as a % of spontaneous alternations by total number of possible alternations. All values from 12-month-old female mice that completed at least 5 entries (*n* = 5-10) were quantified as mean ± *SEM*. * One-way ANOVA with pairwise post-hoc Fisher LSD test p-value < 0.05. (B) Number of Y-maze arm entries. All values from 12-month-old female mice that completed at least 5 entries (*n* = 5-10) were quantified as mean ± *SEM*. * One-way ANOVA with pairwise post-hoc Fisher LSD test p-value < 0.05. (C) Spontaneous alternations normalized by distance traveled. Only mice that completed 5 entries or more were considered. Data was quantified as mean ± *SEM* (*n* = 5-10, 12-months-old female mice). One-way ANOVA with pairwise p-value calculated via post-hoc Fisher LSD test.

**
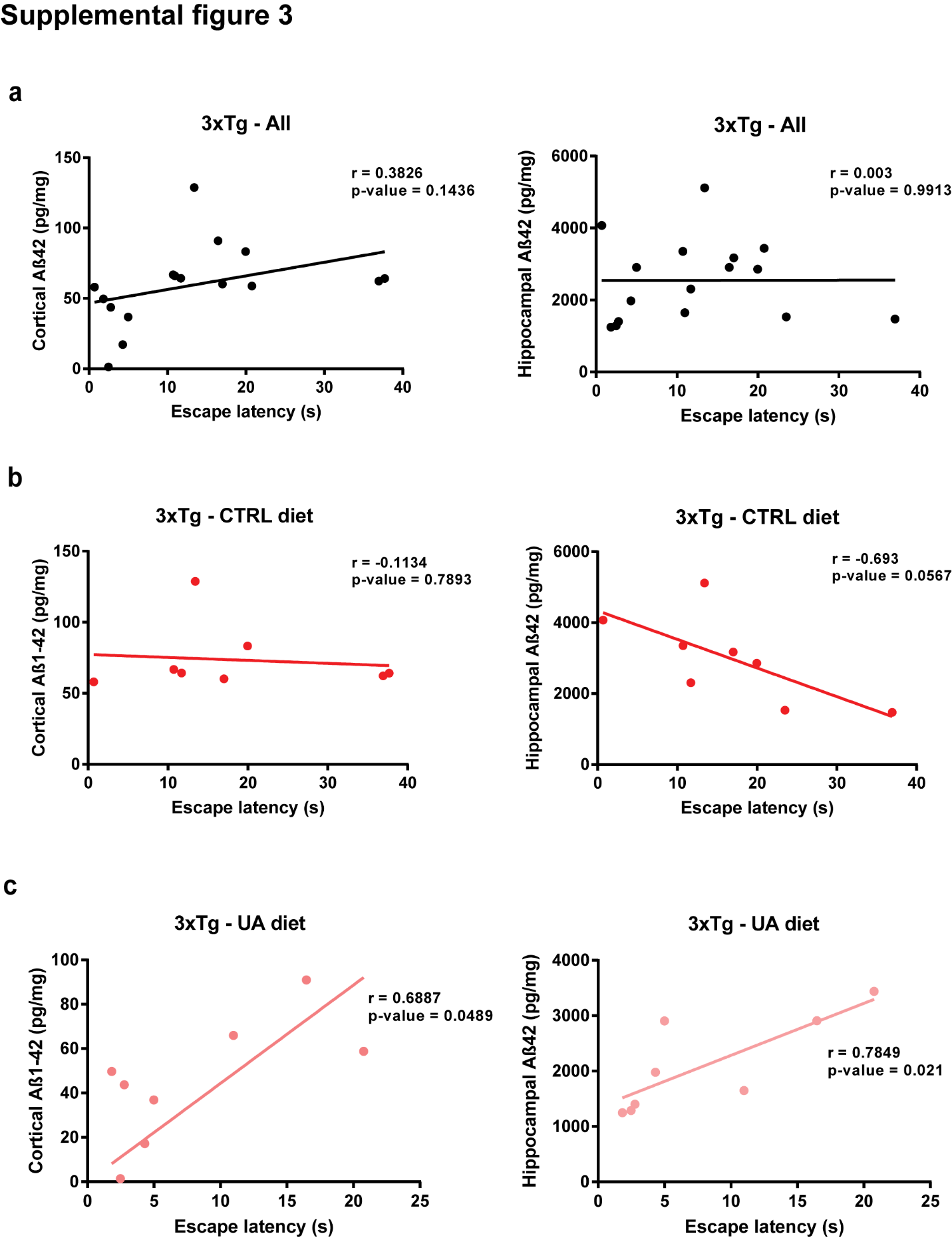
**

**Fig. S3** Aß42 concentration in the cortex and hippocampus correlates with spatial learning and memory in 3xTg/UA mice but not in 3xTg/CTRL mice. Similar to Fig. 4, analysis of the linear correlation between Aß42 concentration in cortical and hippocampal homogenates by sandwich ELISA and spatial learning and memory measured with escape latency in the MWM task in (A) all 3xTg-AD mice (n=16), (B) 3xTg/CTRL mice (n=8) or (C) 3xTg/AD mice (n=8).


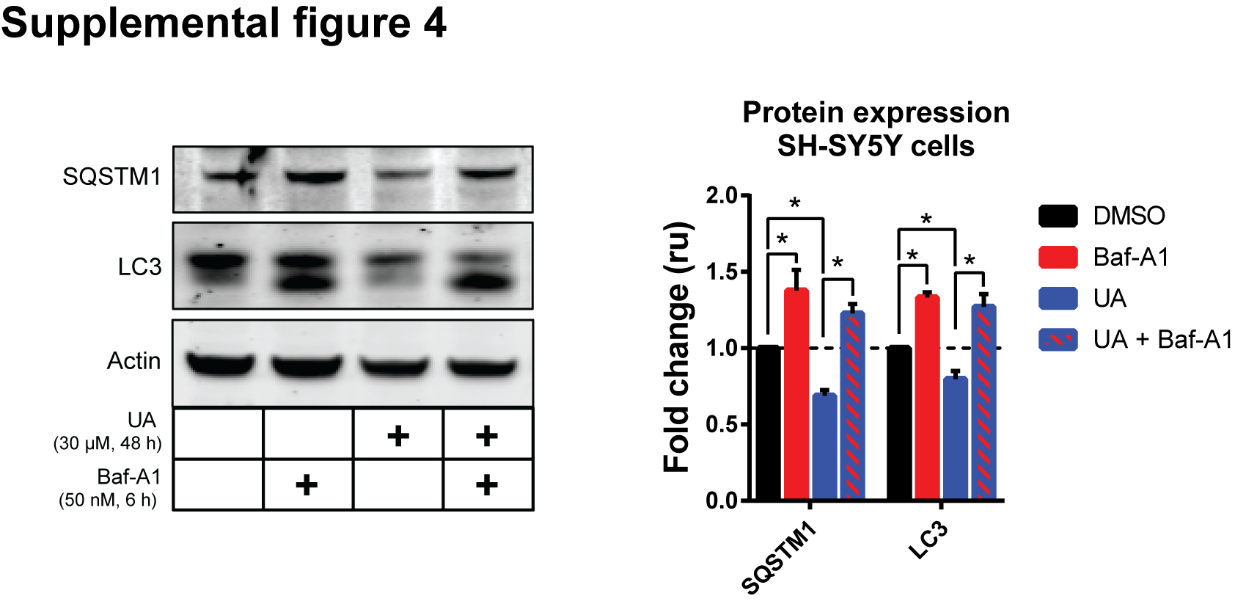


**Fig. S4** UA enhances autophagy flux in SH-SY5Y cells. Western blot analysis of SQSTM1 (p62) and LC3B protein levels, using specific antibodies in SH-SY5Y cells treated with or without UA and/or bafilomycin. Shown is a representative figure for four independent experiments. Quantification of changes in protein expression is presented as mean fold change in relative units (ru) ± *SEM* by normalizing each protein’s band intensity to the expression of actin and are presented as a fold-change relative to the samples treated with DMSO as vehicle. * p-value < 0 .05 calculated via unpaired t-test.

**
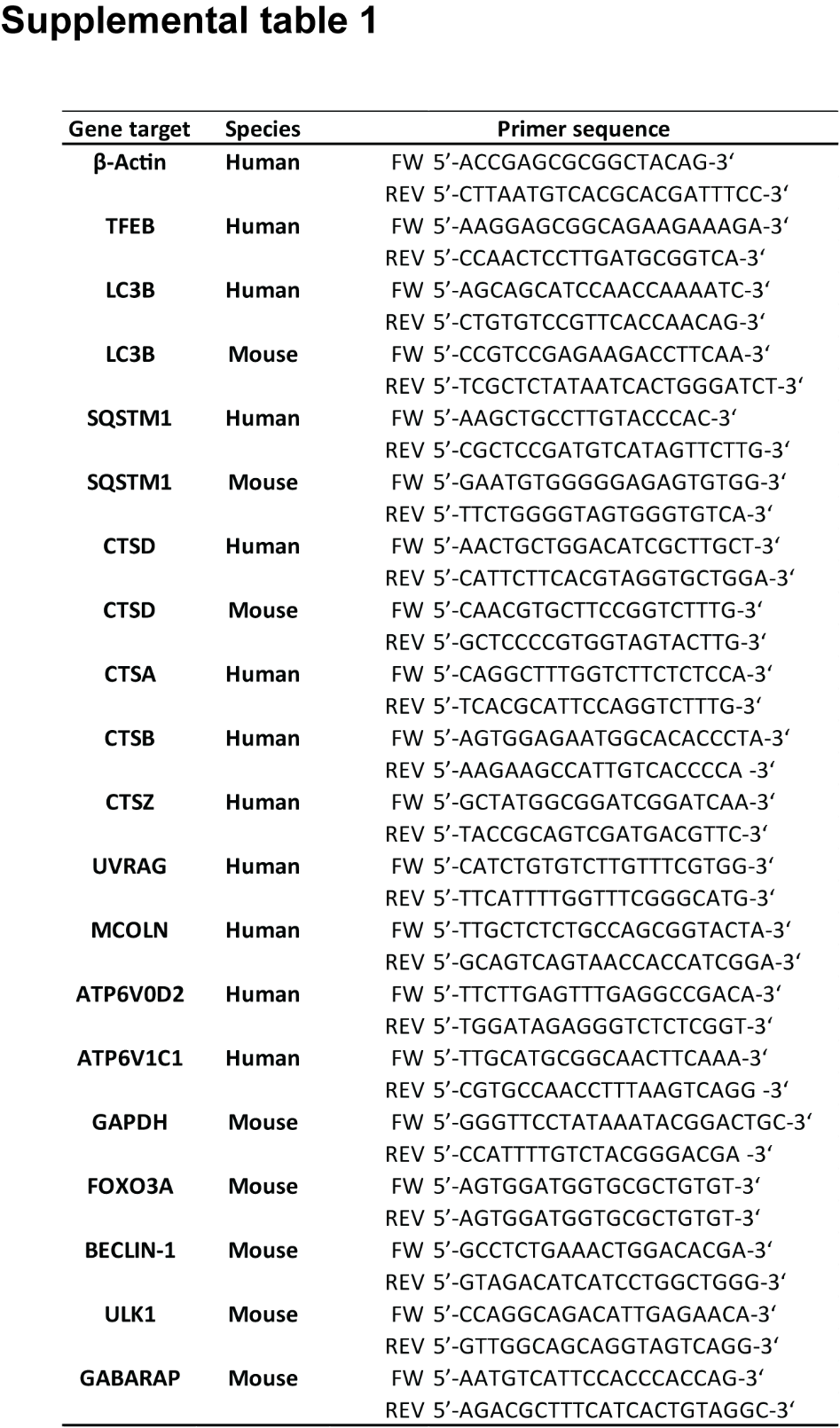
**

**Table S1** Gene-specific primers used for RT-qPCR
